# The London University

**Published:** 1829

**Authors:** 


39. *The London University.*—Many of our friends may not be aware, that London, which most Americans regard as an emporium of letters and science, not less than politics, fashion and trade, has never had a University, for literary, scientific, or *medical* instruction. Nevertheless, the number of students of physic and surgery annually congregated, in that immense city, has not been small; but they have been drawn thither by the undefinable attractions which a great capital exerts; by the number and extent of its public hospitals; and by the merited renown of its physicians, surgeons, obstetricians, and pharmacopolists. It had long been perceived, however, that an *organised* faculty—a confederacy of teachers—was a *desideratum* in the metropolis; but difficulties, into which it is not our purpose to inquire, prevented, till lately, the consummation so earnestly desired, by the enlightened and liberal minded portion of our British brethren.

The London University, for some time a mere project, has at length been *established;* and the first session of its Medical Department, commenced with the month of October, 1828. In the last number of the Medico-Chirurgical Review, (January, 1829) we find an ample and interesting account of the initial series of introductory lectures. But two of its seven professors are well known to the profession in America, and they are both Scotchmen. Whether any more are from the 'Northern hive' of poets, critics, metaphysicians and physicians, we are unable to say.

The Faculty consists of

Mr. Charles Bell, *Surgery.*
Dr. Granville Sharpe Pattison, *Anatomy.*
" Conolly, *Institutes and Practice.*
" Davis, *Obstetrics.*
" Watson, *Clinical Medicine.*
" Thompson, *Materia Medica.*
" Turner, *Chemistry.*

The Editor is liberal in his applause of each introductory lecture; and does not hesitate to predict, for the new institution, the most brilliant success. The name of Charles Bell, alone, would inspire confidence on this side of the water; while that of Mr. Pattison, the same gentleman who lately filled the chair of Anatomy in the University of Maryland, affords no inconclusive evidence of sound eclectic views in the Regents of the University. Dr. Thompson, Dr. Turner and Dr. Davis (the first especially) are advantageously known in this country; but of Dr. Conolly and Dr. Watson we know but little, and theirs are certainly not the names, which *a priori*, we should have expected to see placed before the important professorships of theoretical and practical medicine. The Editor, however, speaks of them in such language as compels us to believe, that the medical department of the new, or as he calls it, the People's University, must speedily attain to a high rank among the most celebrated schools, which so pre-eminently distinguish the nineteenth century. The number of pupils already entered was said to be very great—indeed three times as great as was anticipated.

The time was, when such information as we now communicate, would have been of practical advantage to the students of America, but that era, happily, has passed away, never to return. We no longer look to Europe, for the education of the business and working members of the profession. The most numerous and useful, if not the most accomplished physicians and surgeons of the United States, are now educated at home, never visiting the old world in any stage of their studies; and, although many of them are *badly* educated, there is little real necessity for going abroad. The brevity and laxness of study, to which their defects may be fairly ascribed, once corrected, they would become profound and elegant practitioners, without foreign assistance; while left uncorrected, all the vaunted opportunities of Paris, Edinburgh and London, would be lost upon them.

